# Beyond Oral Therapies: A Practical Approach to Overcoming Barriers to Intracavernosal Injection Therapy in Clinical Practice

**DOI:** 10.1590/S1677-5538.IBJU.2026.0409

**Published:** 2026-07-02

**Authors:** Lucas Vasconcelos Sanvido, Nilson Marquardt, Thiago Afonso Teixeira, Felipe Carneiro, Bruno Chiesa Gouveia Nascimento, Jorge Hallak

**Affiliations:** 1 Faculdade de Medicina da Universidade de São Paulo Hospital das Clínicas Departamento de Cirurgia São Paulo SP Brasil Departamento de Cirurgia, Divisão de Urologia, Hospital das Clínicas, Faculdade de Medicina da Universidade de São Paulo - HC-FMUSP, São Paulo, SP, Brasil; 2 Universidade Federal do Amapá Hospital Universitário Departamento de Cirurgia Macapá AP Brasil Divisão de Urologia, Departamento de Cirurgia, Hospital Universitário, Faculdade de Medicina, Universidade Federal do Amapá UNIFAP, Macapá, AP, Brasil; 3 Universidade de São Paulo Instituto de Estudos Avançados Grupo de Estudos em Saúde Masculina São Paulo SP Brasil Grupo de Estudos em Saúde Masculina, Instituto de Estudos Avançados, Universidade de São Paulo - USP, São Paulo, SP, Brasil; 4 Instituto Androscience para Ciência, Educação e Projetos Avançados em Saúde Masculina Laboratório de Andrologia Clínica e de Pesquisa de Alta Complexidade Centro de Ciência e Inovação em Andrologia São Paulo SP Brasil Androscience, Centro de Ciência e Inovação em Andrologia e Laboratório de Andrologia Clínica e de Pesquisa de Alta Complexidade e Instituto Androscience para Ciência, Educação e Projetos Avançados em Saúde Masculina, São Paulo, SP, Brasil; 5 Faculdade de Medicina da Universidade de São Paulo Unidade de Toxicologia Reprodutiva Departamento de Patologia São Paulo SP Brasil Departamento de Patologia, Unidade de Toxicologia Reprodutiva, Faculdade de Medicina da Universidade de São Paulo - FMUSP, São Paulo, SP, Brasil

**Keywords:** therapy [Subheading], Erectile Dysfunction, Treatment Adherence and Compliance

## Abstract

The objective of this manuscript is to present intracavernous therapy to readers, serving as a model and guide for physicians on how to introduce and manage it in their patients. Furthermore, we propose a strategy designed to ensure maximum safety with minor complications and provide an effective implementation of intracavernosal injection (ICI) therapy for the management of erectile dysfunction (ED). While phosphodiesterase type 5 inhibitors (PDE5i) are recognized as first-line therapy, approximately one-third of patients do not achieve satisfactory clinical outcomes, highlighting the importance of ICI as a highly effective second-line option. This comprehensive review details patient selection, drug formulation, structured patient education, stepwise dose titration, management of adverse events, and longitudinal follow-up. This approach is intended to minimize treatment-related complications, enhance patient confidence and adherence, and optimize long-term therapeutic outcomes. By suggesting a standardized clinical application of ICI therapy, this review aims to address key barriers that currently restrict its broader adoption in routine clinical practice.

## INTRODUCTION

Erectile dysfunction (ED), defined as the recurrent or persistent inability to achieve and maintain an erection sufficient for satisfactory sexual performance, is a prevalent condition affecting millions of men globally. The prevalence and severity of ED increase significantly with age, impacting more than 50% of men in older age groups (1-3). Treatment modalities include oral pharmacotherapy, vacuum devices, and penile prostheses (4, 5).

Phosphodiesterase type 5 inhibitors (PDE5i), such as sildenafil, tadalafil, and vardenafil, are considered first-line therapies due to their established efficacy, ease of administration, and favorable safety profiles (6). These agents enhance the nitric oxide–cyclic guanosine monophosphate (NO–cGMP) pathway by inhibiting PDE5, thereby promoting smooth muscle relaxation and increasing penile arterial inflow during sexual stimulation (7). However, up to 30–40% of patients do not achieve satisfactory erectile responses, being candidates for 2nd and 3rd line therapies. Additionally, PDE5i are contraindicated in patients taking nitrates, those with unstable cardiovascular disease, or those who have experienced recent major cardiovascular events. Adverse effects commonly include headache, flushing, nasal congestion, and dyspepsia (8).

Intracavernosal injection (ICI) therapy involves the direct injection of vasoactive agents into the corpora cavernosa, including prostaglandin E1, phentolamine, and papaverine, administered individually or in combination (called bimix or trimix) (9, 10). It is an effective second-line treatment, with reported success rates exceeding 85%, even among PDE5i non-responders (7, 11). Despite that, it is striking that the regular use of ICI therapy is underrated and underused by healthcare professionals who deal directly with patients with ED, frequently skipping directly to penile prosthesis after failure of PDE5i therapy, precluding patients from the chance of trying this less-invasive therapy.

Reasons for the underutilization of such an effective tool include restricted access to medical education and proper physicians’ training on how to reduce the risk of adverse events, strategies to initiate and titrate the dosage, as well as patients’ training to overcome psychological and objective difficulties in self-administration, needle-related anxiety, logistical challenges related to drug handling and storage, and high cost (12, 13).

This review outlines the main concepts behind ICI, pharmacological agents, proper patient selection, and training. The purpose is to share an institutional protocol, developed and tested over years of use in a Tertiary Care Hospital, to address limitations that currently restrict its broader clinical adoption and to establish a safe, standardized, and practical approach to ICI therapy.

## MATERIALS AND METHODS

A narrative review format was employed to educate readers on the central concepts of ICI therapy, while providing clinicians with a pragmatic, evidence-based framework for its safe and effective application in real-world clinical practice. Rather than systematically aggregating all available data, the review prioritized key studies and influential publications that have significantly shaped the development and current use of ICI therapy, as well as our own experience.

A targeted literature search was conducted in the PubMed database in August 2025, covering studies published during the previous 25 years. The search strategy utilized the Medical Subject Headings (MeSH) term "Intracavernosal Injection Therapy" in combination with the keywords "Self-Injection Therapy for Erectile Dysfunction." Bibliographies of selected articles were also manually reviewed to identify additional relevant references.

Due to the descriptive nature of the review, neither quantitative data synthesis nor formal assessment of methodological bias was performed. Article selection was based on clinical applicability and the relevance of findings to real-world practice.

We present an institutional clinical strategy for ICI therapy implementation at a tertiary referral center. The protocol was developed through integration of contemporary evidence, structured consensus among experienced sexual medicine urologists, and retrospective evaluation of routine clinical outcomes. It standardizes key aspects of care, including drug selection, patient training, stepwise dose titration, and management of treatment-related complications. This structured approach is designed to help clinicians safely initiate and optimize ICI therapy to improve efficacy and long-term adherence. No formal external validation was conducted.

## RESULTS AND DISCUSSION

As previously mentioned, ICI therapy may be administered as monotherapy or combination therapy (bimix, trimix, or quadrimix) to promote erection. Each pharmacological compound has distinct mechanisms of action and potential adverse effects, and combining them enables a multi-mechanistic approach, enhancing synergistic efficacy while allowing lower individual drug concentrations. This strategy may improve tolerability and reduce the incidence of dose-dependent adverse events associated with higher single-agent doses (9). The four vasoactive agents for ICI combinations are prostaglandine, papaverine, phentolamine, and atropine

## AGENTS AND SOLUTIONS

### Prostaglandin E1 - (PGE1)

The use of prostaglandin E1 for erectile dysfunction has been described since 1986 (14). In recent decades, prostaglandin E1 has become the most extensively studied intracavernosal vasoactive agent, being the most common agent in monotherapy.

The PGE1 mechanism of action involves stimulating adenylate cyclase and locally blocking alpha-1 receptors, thereby promoting smooth muscle relaxation and arterial dilatation. The effective dose varies with the severity of the ED and its etiology, ranging from 2.5 μg to 40 μg when used as monotherapy. The most common monotherapy dose is between 10 and 20 μg (15). This agent is commercially available in a lyophilized formulation, marketed under the brand name Caverject® (Pfizer), and can be obtained through standard retail pharmacies with a prescription.

The most frequent adverse effect of prostaglandin injections is penile pain, particularly at doses exceeding 15μg. Additionally, once reconstituted into active form—or when incorporated into compounded solutions such as trimix—the medication must be stored at 2-8°C (36–46°F) and has a stability period of up to 30 days..

### Papaverine

Papaverine was the first pharmacological agent identified as effective for treating erectile dysfunction. Discovered in 1848 as an opium alkaloid from Papaver somniferum, a plant, papaverine is frequently used in combination with other vasoactive agents in ICI therapy due to its adverse effect profile. Its mechanism of action includes phosphodiesterase inhibition, increased cAMP levels, reduced smooth muscle contraction, and promotion of arterial dilatation (16).

Papaverine monotherapy is typically administered at doses ranging from 10 to 60 mg (17). One of the most significant agent-specific adverse effects is penile fibrosis, which is thought to be related to the solution's acidic nature. Regarding penile fibrosis, Lakin et al. reported in a study of 100 men receiving ICI therapy with papaverine, with or without phentolamine, for 29 months, that nodules or plaques were associated with penile fibrosis. Nodules were defined as round areas at the injection site without other symptoms and were observed in 9 patients, typically resolving spontaneously and not being associated with penile curvature. Plaques occurred in 6 patients, further from the injection site, were indistinguishable from Peyronie's plaque and were, in some cases, associated with significant penile deformity, interfering with intercourse (18).

Although this cohort of men with severe ED lacked a control group to compare the rate of spontaneous Peyronie's disease development and establish causality, there remains a reluctance to use papaverine as monotherapy. Combination therapy with other vasoactive agents allows lower total papaverine doses, potentially reducing the risk of dose-related adverse effects (19).

### Phentolamine

Phentolamine's vasoactive function was discovered in 1978. The mechanism of action is a reversible, competitive antagonist at alpha-adrenergic receptors. It causes vasodilation of vascular smooth muscle and reduces the adverse sympathetic effects on erection (20).

Phentolamine is primarily used as an adjuvant in combination with other vasoactive agents, as its efficacy as a single agent is limited, even at higher doses (15).

### Atropin

The use of atropine in erectile dysfunction has been reported by Virag et al. since 1985 (21). Atropine at low concentrations (10^-8 M) blocks muscarinic receptors and regulates neurogenic corporal smooth muscle relaxation. In contrast, higher concentrations (10^-3 M) may release endothelium-derived relaxing factor, a neurotransmitter involved in penile erection (22). Atropine should not be used as a single agent; typical doses range from 0.075 mg/mL to 0.15 mg/mL of atropine sulfate, most commonly in combination with other vasoactive agents, particularly in the quadrimix formulation (23). Figure-1 summarizes the mechanism of penile erection and the pharmacological action of intracavernosal agents.

**Figure 1:**
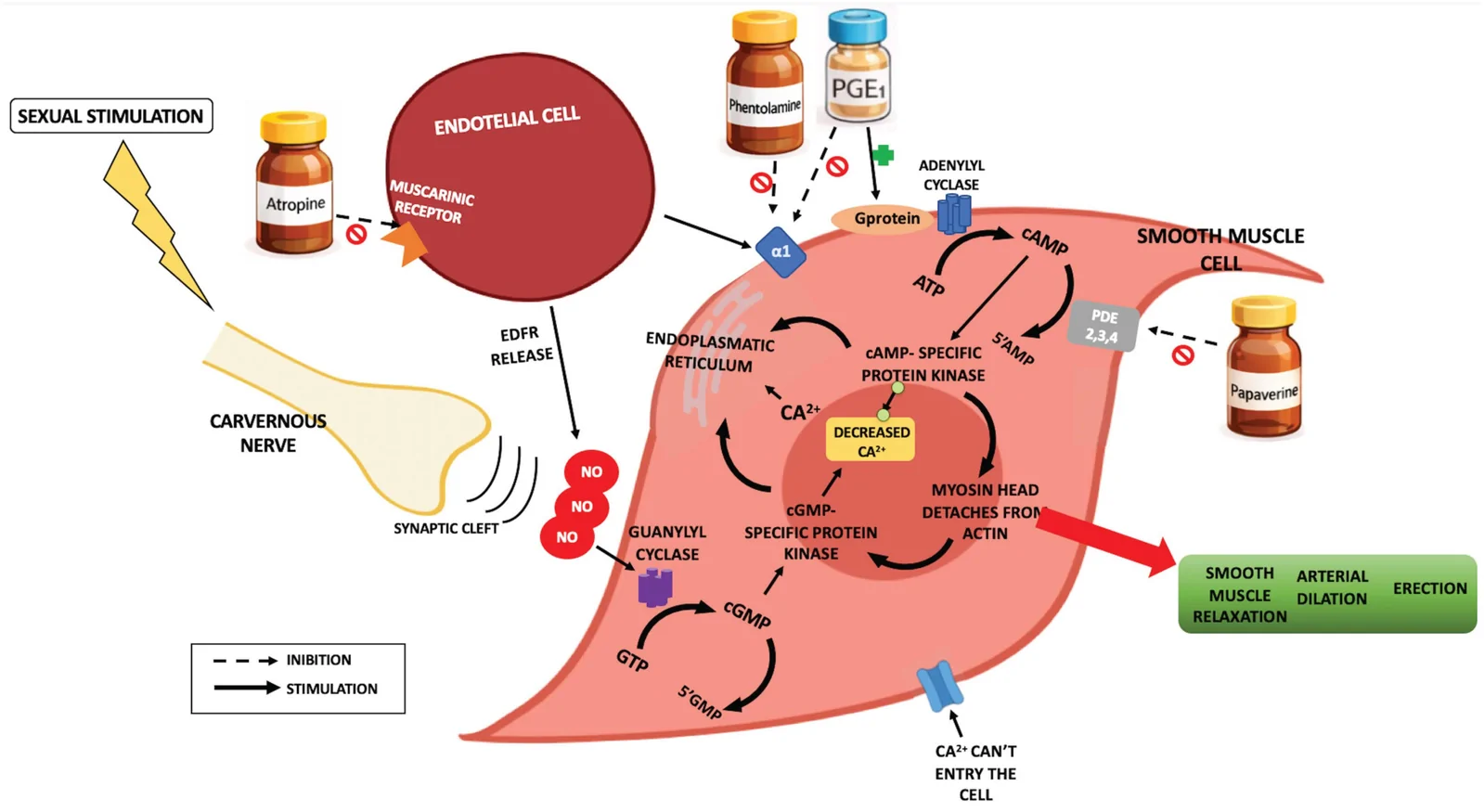
Mechanisms of penile erection and pharmacological action of intracavernosal agents.

Diagram illustrates the intracellular pathways regulating penile smooth muscle tone and the principal sites of action of commonly used intracavernosal pharmacotherapies. Sexual stimulation activates parasympathetic signaling, promoting nitric oxide (NO) release from endothelial cells via endothelium-derived relaxing factor (EDFR). NO stimulates guanylyl cyclase, leading to increased cyclic guanosine monophosphate (cGMP) production. Cyclic adenosine monophosphate (cAMP) and cGMP, the key intracellular second messengers mediating smooth muscle relaxation, activate their respective protein kinases. These kinases phosphorylate downstream target proteins, resulting in potassium channel opening, calcium channel inhibition, and sequestration of intracellular calcium by the endoplasmic reticulum. The consequent reduction in intracellular calcium (Ca^2+^) levels promotes smooth muscle relaxation, arterial dilation, and erection. Prostaglandin E1 (PGE1) activates adenyl cyclase via G protein–coupled receptors, increasing cAMP levels and further contributing to Ca^2+^ reduction and smooth muscle relaxation. Papaverine acts as a nonspecific phosphodiesterase inhibitor, enhancing cyclic nucleotide accumulation. Phentolamine is a competitive α-adrenergic receptor antagonist that attenuates sympathetic-mediated smooth muscle contraction. Solid arrows represent stimulatory pathways, whereas dashed arrows indicate inhibitory effects. GTP denotes guanosine triphosphate; ATP denotes adenosine triphosphate.

### Combination

The nomenclature for combinations of vasoactive drugs is based on the number of vasoactive agents in the formula, not on their concentrations. Below are some examples of commercially available formulations in compounding pharmacies in our country:

Bimix – a combination of two vasoactive agents (e.g., Papaverine 30mg/mL + Phentolamine 1mg/mL; Prostaglandin 20 mcg/mL + Phentolamine 2mg/mL).

Trimix – a combination of three vasoactive agents (e.g., Papaverine 30mg/mL + Phentolamine 1mg/mL + Prostaglandin 10mcg/mL) * most referred formula in the literature.

"Super" Trimix - higher concentration of three vasoactive agents (e.g., Papaverine 25mg/mL + Phentolamine 4mg/mL + Prostaglandin 20mcg/mL).

Quadrimix – a combination of four vasoactive agents (e.g., Papaverine 20mg/mL + phentolamine 3,3mg/mL + prostaglandin 44mcg/mL + Atropine 0,11mg/mL).

Combinations and dosages of these agents vary by pharmacy and can be selected based on the patient's response, side effects, and preferences. The effective dose represents the effect of available agents, their concentration in a given solution, and, of course, the injected volume. Accordingly, dosing discussions should focus on the delivered pharmacological dose rather than volume alone. To standardize communication and reduce ambiguity, the use of international units (IU)—analogous to insulin syringes—is recommended, with 1 IU corresponding to 0.01 mL.

Clinical efficacy should be interpreted as a function of the administered dose in the setting of patient-specific variables, including cavernosal tissue integrity, arterial integrity, veno-occlusive function, and psychological well-being. Erectile response, therefore, reflects a dynamic interaction between pharmacologic stimulation and biologic capacity, while a stressful environment and consequent adrenergic influence will act negatively.

### Adverse Effects

The majority of side effects of the intracavernous injection are related to the puncture site or local complications. Otherwise, the rates of side effects are conflicting in the literature and range from 5% to 94% (24–26).

### About starting the treatment for erectile dysfunction

A comprehensive evaluation of the patient's sexual history, emphasizing a patient-centered approach, is essential. As with all patients with ED, this assessment should include a thorough medical, sexual, and psychological history, a physical examination, and basic laboratory and hormonal testing (7). Patients should be informed about the association between ED, endothelial dysfunction, and cardiovascular disease. If appropriate, according to cardiovascular risk stratification (27), complementary investigations must be conducted (28). Education regarding lifestyle modifications, such as dietary changes and increased physical activity, can improve overall and sexual health. A detailed history of ED, previous treatments, including previous exposure to ICI, such as in previously performed penile duplex doppler with induced erection, should be inquired, and alignment of patient expectations is fundamental to effective management (29). Patients with contraindications, failure to respond, or intolerance to PDE5i are candidates for ICI therapy.

### The Patient Selection

Shared decision-making, incorporating a comprehensive assessment of patient needs, comorbidities, and potential barriers to treatment, is essential to optimize outcomes, reduce dropout rates, and minimize adverse events.

Although there are generally no absolute contraindications to intracavernosal injection (ICI) therapy—making it a feasible option for most patients (30)—careful patient selection remains critical. Evaluation should include manual dexterity, tremor, visual acuity, penile exposure, abdominal circumference, and the ability to perform self-injection or to receive assistance from a partner (31), as these factors may significantly impact treatment adherence and effectiveness.

Additionally, needle anxiety, substance abuse, and psychiatric disorders may represent important limitations to therapy. In this context, partner involvement in injection training may help mitigate some of these barriers and improve overall treatment adherence.

### Contraindications

Contraindications to intracavernosal therapy primarily involve hypersensitivity to vasoactive agents or conditions that elevate the risk of priapism, fibrosis, or bleeding. The literature identifies absolute contraindications as a history of priapism, hemoglobinopathy, severe penile fibrosis, penile implants, and bleeding diathesis(9, 15, 17, 31, 32).

However, some clinical experiences suggest these may not always constitute absolute contraindications. Intracavernosal injection therapy has been used in patients receiving antiplatelet agents, such as acetylsalicylic acid (ASA), and anticoagulants with an overall favorable safety profile. Available data indicate that the main concern in this population is an increased risk of minor bleeding complications, including ecchymosis or hematoma at the injection site, rather than major hemorrhagic events (32). In this context, pragmatic measures derived from clinical practice—such as rigorous patient training in injection technique, use of the smallest effective needle (0,30 x 8mm), proper site selection, and prolonged local compression after injection—may help mitigate bleeding risk. With appropriate patient selection and adherence to these precautions, ICI can be performed safely in many individuals, and current evidence does not support considering antithrombotic therapy as an absolute contraindication. However, caution remains warranted (7, 17, 33). It remains essential to inform patients about potential complications and to engage in shared decision-making (30).

### Injection training

Adequate training for safe and confident self-administration of ICI therapy is essential. The initial administration must be performed in the clinical setting by a physician assistant or a trained nurse under a urologist's supervision (5, 7, 15, 17, 34).

The primary goal of the initial visit is not to repeatedly administer injections in the office until the ideal dose is reached, but rather to familiarize the patient with the entire therapeutic process. This includes preparing the syringe, understanding the use of international units (IU) as the standardized language for dose communication, anatomical orientation, proper penile positioning for injection, and experience with the first intracavernosal injection (17). During this first visit, a standardized low initial dose, selected based on the individual patient's clinical profile, is administered in the office. This dose serves both as a safety measure and as a baseline assessment of pharmacologic responsiveness.

At the second visit, the patient is expected to independently prepare the medication and perform the injection under the supervision of the training team. At this stage, adjustments - including modification of the drug formulation and/or the administered volume - may be made based on the response observed in the initial visit, always balancing efficacy and safety.

Although many patients are discharged after the second supervised injection and begin home administration, the optimal therapeutic dose is often achieved progressively over the first months of treatment. Ongoing communication between the patient and the prescribing team is essential to allow careful dose titration, minimize adverse effects—particularly prolonged erections—and ensure long-term adherence and confidence with therapy.

Comprehensive counseling regarding potential complications is mandatory. Patients must commit to strict adherence to prescribed dosing, maintain direct access to the medical team for guidance, and have clear instructions on how to seek emergency care in the event of adverse outcomes such as prolonged erections or priapism.

Nowadays, two injection delivery systems are available through compounding pharmacies in our country. The first—commonly used worldwide—consists of a multidose vial and a disposable syringe. In this method, the patient must aspirate the prescribed dose of the vasoactive agent from the vial and administer it using a disposable syringe and an insulin needle. In our practice, we use the 0,30 x 8mm needle. It is essential to verify the syringe calibration, as some are marked in international units (IU), while others are calibrated in milliliters (mL) or cubic centimeters (cc).

The second system comprises medication cartridges for use with injection pens. This format has gained popularity due to its numerical dose display in international units, which facilitates dose titration and enhances patient confidence.

In this system, disposable needles are attached to the pen before administration, eliminating the need for manual dose preparation once the medication is in the cartridge. Consequently, patients can focus primarily on proper injection technique rather than dose measurement, simplifying the procedure and potentially reducing dropout rates (31, 35).

Before the injection, it is essential to clean the area with alcohol swabs, then stretch the penis by the glans and insert the needle completely, in the dorso-lateral of the penile shaft at a 90° angle to avoid missing injections and perform an adequate and safe intracavernous administration (15). It is important to instruct patients to administer the injection laterally at the 3 o'clock or 9 o'clock position, while avoiding blood vessels, and always alternate the puncture side. Immediately after the injection, the puncture site should be compressed for 30 to 60 seconds. It's not recommended to compress or use a tourniquet at the base of the penis (17). Figures 2 and 3 illustrate the technique for intracavernosal injection.

**Figure 2:**
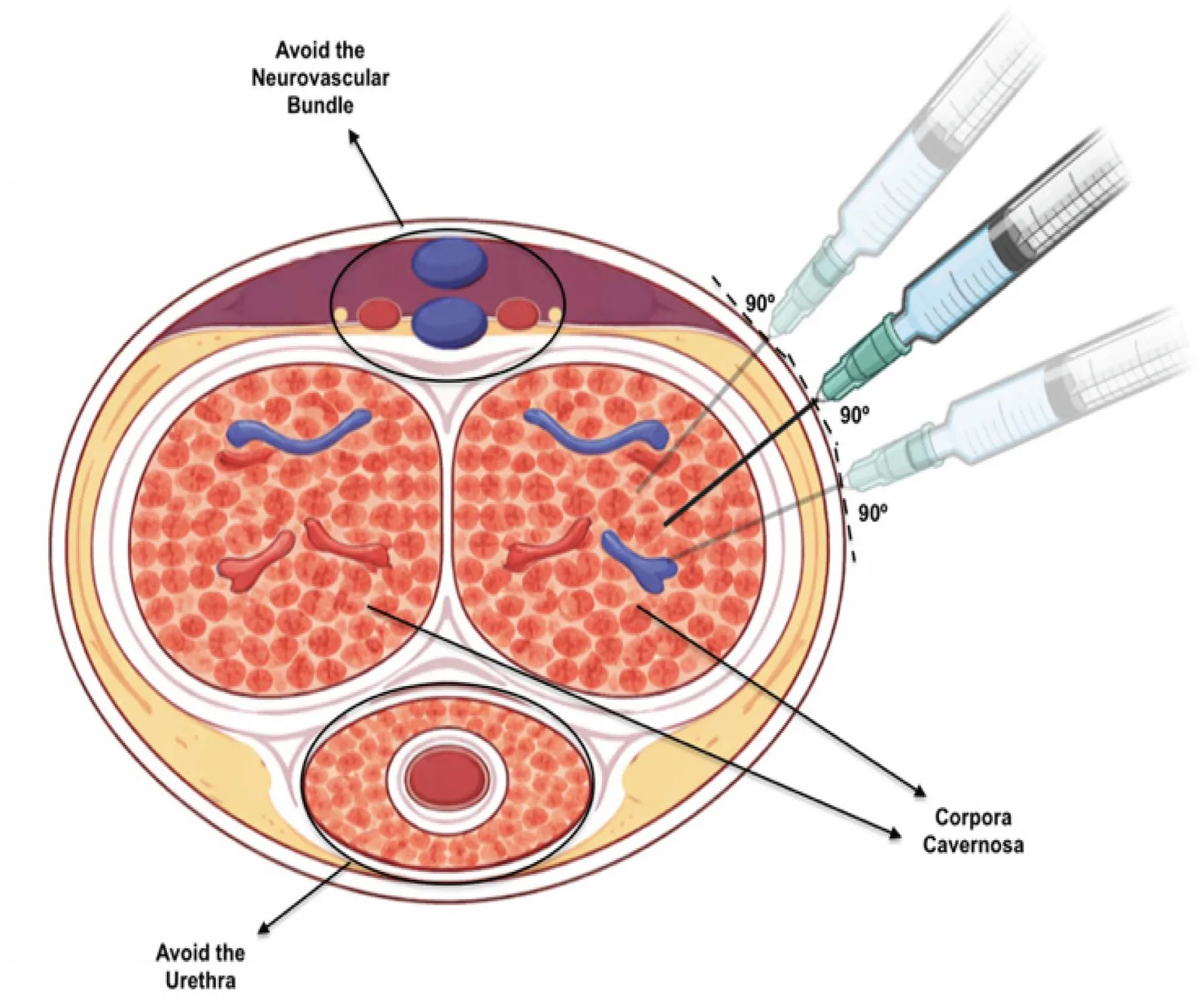
Transversal anatomy of the penis and recommended intracavernosal injection technique.

Cross-sectional representation of the penile shaft demonstrating the corpora cavernosa as the target structures for intracavernosal drug administration. Safe needle placement is performed at the mid-shaft level at the 10 o'clock (left lateral) or 2 o'clock (right lateral) position, using a perpendicular (90°) angle relative to the penile surface. The dorsal neurovascular bundle and urethra must be avoided during injection. Drug delivery is illustrated using a 29-gauge, ½" needle on a 100-unit syringe, consistent with standard intracavernosal injection practice.

**Figure 3:**
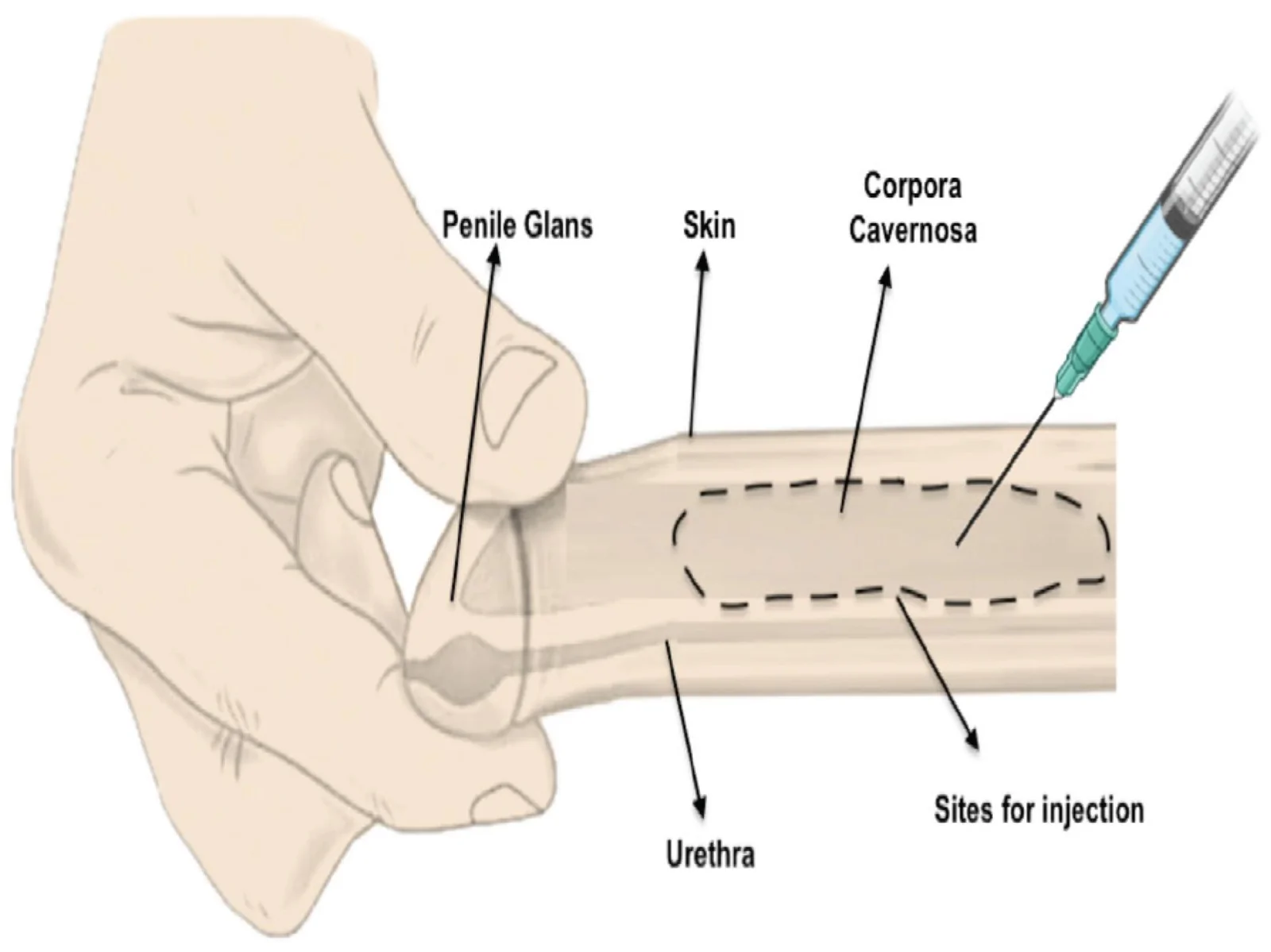
Schematic representation of intracavernosal injection.

Illustration of the penis demonstrating the relationship between the corpora cavernosa, corpus spongiosum, urethra, and penile glans. The recommended intracavernosal injection sites are illustrated within the cavernosal bodies, emphasizing proper needle placement. This diagram highlights the structural basis underlying safe intracavernosal pharmacotherapy.

### Choosing a vasoagent and starting dose

After determining that a patient is a candidate for intracavernous therapy, titration of the dose and selection of an appropriate vasoactive agent should consider the etiology of erectile dysfunction, risk factors, comorbidities, previous failed attempts, and residual erectile function. During this phase, the risks of prolonged erections and insufficient dosing are elevated, underscoring the importance of initiating treatment in a clinical setting under urologist supervision (29, 36).

The preferred vasoactive agents are typically Bimix (Papaverine 30 mg/mL and Phentolamine 1 mg/mL) or Trimix (Papaverine 30 mg/mL, Phentolamine 1 mg/mL, and Prostaglandin 10 mcg/mL). For organic erectile dysfunction, the Trimix dose should be determined based on previous erectile response or erection quality. PDE5i should also be considered. For neurogenic erectile dysfunction, a low dose of Bimix is recommended.

In patients following radical prostatectomy, with normal pre-procedure erectile function, a low dose of Bimix is advised. For patients with reduced pre-procedure erectile function after radical prostatectomy or those who have undergone pelvic irradiation, a low dose of Trimix is initially recommended (29).

If the patient has previously undergone Penile Doppler Duplex Ultrasonography (PDDU) with an adequate redosing protocol and achieved a Best Quality Erection or an Erection Hardness Score (EHS) of 3 or greater, the recommended initial dosage for training should be the exam dose or 20% lower to mitigate the risk of prolonged erections. This dosage reduction accounts for the anxiety and adrenergic response associated with PDDU, which can diminish the vasoactive response (30). The initial dosing should be tailored to the patient's current erectile function and the response to PDE5i. Dosing categories are defined as low (1–10 units), moderate (11–50 units), and high (>50 units).

### Dose adjustment

Dose adjustments should be guided by the hardness and duration of the erection achieved after the injection. The primary goal is to achieve an EHS of ≥ 3 or a numerical score of 7 out of 10, indicating an erection sufficient for vaginal intercourse, with a duration of approximately 60 minutes (30). Table-1 provides a suggested safe dose-titration protocol.

**Table 1:** Dose adjustment algorithm for intracavernosal injection therapy.

DOSE	RIGIDITY	DURATION	ADJUSTMENT
1-5 UNITS	≤ 20%	Irrelevant	Increase 5 units
1-5 UNITS	30-50%	Irrelevant	Increase 5 units
1-5 UNITS	60-70%	≤ 15 minutes	Increase 2 units
1-5 UNITS	60-70%	15-60 minutes	Maintain dose
1-5 UNITS	60-70%	> 60 minutes	Reduce 1 unit
1-5 UNITS	> 70%	≤60 minutes	Maintain dose
1-5 UNITS	> 70%	> 60 minutes	Reduce 2 units
6-10 UNITS	≤ 20%	Irrelevant	Increase 5 units
6-10 UNITS	30-50%	Irrelevant	Increase 5 units
6-10 UNITS	60-70%	≤ 15 minutes	Increase 2 units
6-10 UNITS	60-70%	15-60 minutes	Maintain dose
6-10 UNITS	60-70%	> 60 minutes	Reduce 2 units
6-10 UNITS	> 70%	≤60 minutes	Maintain dose
6-10 UNITS	> 70%	> 60 minutes	Reduce 2 units
11-30 UNITS	≤ 20%	Irrelevant	Increase 5 units
11-30 UNITS	30-50%	Irrelevant	Increase 5 units
11-30 UNITS	60-70%	≤ 15 minutes	Increase 2 units
11-30 UNITS	60-70%	15-60 minutes	Maintain dose
11-30 UNITS	60-70%	> 60 minutes	Reduce 2 units
11-30 UNITS	> 70%	≤ 60 minutes	Maintain dose
11-30 UNITS	> 70%	> 60 minutes	Reduce 2 units

Recommended dose adjustment strategy based on erectile rigidity and duration of erection following intracavernosal injection. Dose modifications are expressed in units, where 1 unit corresponds to 0.01 mL (0.01 cc). The algorithm is based on a standard trimix formulation containing papaverine 30 mg/mL, phentolamine 1 mg/mL, and prostaglandin E1 (alprostadil) 10 μg/mL.

### Management of adverse events and complications

Reported complication rates are variable and predominantly involve minor injection-site events. Among men who discontinue treatment, penile pain or discomfort is the most commonly reported adverse effect, most frequently associated with prostaglandin doses exceeding 15μg (25).

According to the literature, the prevalence of pain associated with PGE1 ranges from 9% to 34%. According to Sung et al., 45.9% of patients who discontinued with ICI cited pain as the primary adverse event (24). Cost is an additional consideration for the treatment modality and dropout rates. Mulhall et al. reported that 28% of men discontinued treatment due to high cost (25).

To ensure the treatment success, some pain management strategies include reducing the prostaglandin concentration or altering the formulation to exclude this agent. Pain typically decreases over the course of treatment, and slower injection rates are associated with reduced discomfort (24, 25, 37).

Priapism is a particularly concerning adverse effect, as it constitutes a urological emergency that can result in tissue damage, fibrosis, and loss of erectile function. The risk of priapism varies by vasoactive agent formulation, but it is mostly determined by the dose used. Priapism is most commonly observed during the initial dose-titration phase, with reported rates ranging from 2% to 15% (36). A prolonged erection is defined as one that persists for between two and four hours, whereas priapism refers to an erection lasting more than 4 hours. Ischemic priapism (low-flow, veno-occlusive) constitutes a medical emergency due to the risk of corporal fibrosis and irreversible erectile dysfunction if not treated promptly. Several conservative treatments have been described, including exercise, ejaculation, ice packs, cold baths, and cold-water enemas (38). The success rates for these measures alone are variable and infrequently reported (39); however, they should be attempted, particularly in patients without immediate access to drainage or injection of a sympathomimetic agent.

For priapism treatment, the first step is injection of a sympathomimetic agent (phenylephrine, etilephrine, or adrenaline) into the corpora cavernosa. Etilephrine is often selected in our country due to the limited availability of phenylephrine. Repeat injections may be administered as needed, and treatment is discontinued once detumescence is achieved. In the absence of detumescence, corporal aspiration is performed following a penile nerve block. Table-2 summarizes the medical treatment.

**Table 2:** Sympathomimetic agents for the management of prolonged erection and priapism.

Drug	Dose/Instructions for use
Phenylephrine	Intracavernous injection of 200 μg every 3-5 minutes. The maximum dosage is 1 mg within 1 hour.
Etilephrine	Intracavernosal injection at a concentration of 2.5 mg in 1-2 mL of normal saline.
Adrenaline	Intracavernous injection of 2 mL of 1/100,000 adrenaline solution up to five times over a 20-minute period.

Recommended sympathomimetic agents and dosing protocols used for intracavernosal administration in the acute management of prolonged erection and ischemic priapism. Doses reflect commonly reported clinical practice and guideline-based approaches aimed at achieving detumescence while minimizing systemic adverse effects.

A 19-G scalp needle should be introduced into one of the corpora cavernosa to facilitate blood outflow and reduce intracavernous pressure. Although most urologists prefer to place the butterfly needle from the side of the shaft, insertion through the glans has been reported to reduce the risk of ecchymosis and hematoma formation (40). Placement of an additional 19-G butterfly needle in the contralateral corpora cavernosa with cold saline irrigation may also be performed (Figure-4) (41, 42). If patients do not respond, shunt surgery is considered the next treatment option (43).

**Figure 4:**
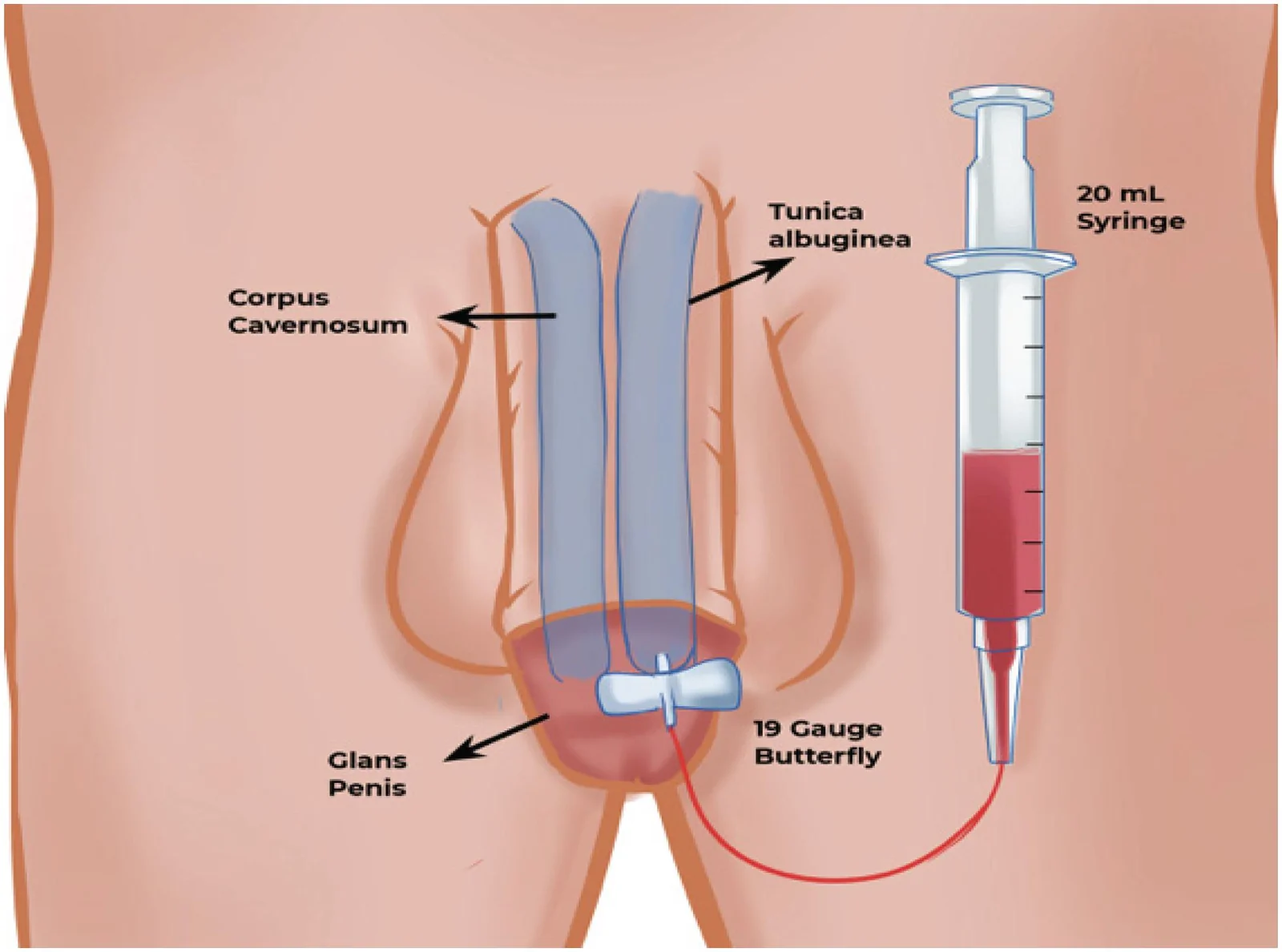
Drainage technique of prolonged erection.

Schematic representation of corporal aspiration performed through the penile glans. A 19-gauge butterfly needle is inserted above the coronal sulcus, in the midglans, into one of the corpora cavernosa. The needle is connected to a 20-mL syringe to aspirate stagnant blood from the corpus cavernosum, facilitating detumescence.

Penile shunt surgery is rarely required to treat ICI priapism. The main objective of the procedure is to create an outflow of ischemic blood from the corpus cavernosum into the corpus spongiosum, thereby restoring normal circulation within these structures (44). Four categories of shunt procedures have been described: percutaneous distal shunts, such as Winter's procedure, Ebbehoj's technique, and T-shunt (45-47); open distal shunts, including Al-Ghorab's procedure and Burnett's technique (48); open proximal shunts, such as Quackles's technique (49), and vein anastomosis shunts, like Grayhack's procedure (50). The choice of shunt procedure may be influenced by surgeon preference and familiarity (42).

In addition to priapism and prolonged erections, the development of plaques and penile curvature is a significant concern. These complications are strongly associated with frequent injections and higher doses of papaverine. Retrospective studies have reported such alterations in 5% to 30% of patients (31), and they account for treatment discontinuation in 24% of cases (24).

Other minor local side effects include nodules at the injection site and small hematomas that do not require intervention. Unusual adverse effects such as inadvertent urethral injection, foreskin swelling, and loss of local sensation have also been reported. In these cases, discontinuation of injections until symptom resolution and reassessment of injection technique are recommended (18, 24). Infection is a possible but rare adverse effect, with only a single case reported in one study (51). Proper injection techniques, single-use of syringes and needles, and strict hygiene at the injection site are essential to minimize this risk.

Systemic side effects are infrequent and mainly include hypotension and dizziness. At higher doses of papaverine, elevations in liver transaminases have been reported, most commonly in patients with a history of alcohol consumption (19, 31).

### Practical Considerations

Several considerations are important to optimize therapeutic outcomes. Penile pain following injection may result from the prostaglandin dose, as some patients exhibit intolerance. Reducing the prostaglandin dose or utilizing a formulation without this vasoactive agent are potential alternatives. Additionally, the requirement to refrigerate prostaglandin can lead to dissatisfaction and increased rates of treatment discontinuation.

Patients with Peyronie's disease or palpable plaques should be informed about the potential risk of exacerbating fibrosis when using papaverine. Consideration should be given to formulations that do not contain papaverine. Patients with coagulation disorders or those receiving anticoagulant therapy are at increased risk for small hematomas and bleeding. Therefore, practitioners should be proficient in injection techniques, and compression should be applied to the injection site for at least 60 seconds in these individuals (30). When the administered dose exceeds 0.5 mL, switching to a more concentrated formulation is recommended (31).

### Follow-up Protocol

Following treatment initiation and dose titration, monthly follow-up is recommended for the first three months. Regular assessment should include erection score, erection duration, administered dose, application technique, and necessary dose adjustments. All patient concerns should be addressed during these visits.

After establishing treatment efficacy and completing the initial follow-up period, office visits every six months are advised. Ongoing evaluation should include monitoring for side effects, penile examination, injection-related difficulties, patient concerns, and overall satisfaction with therapy.

## CONCLUSIONS

Intracavernous injections are highly effective and safe. The implementation of standardized formulations and dosing protocols is essential to optimize outcomes, minimize adverse effects, and reduce therapy discontinuation. ICI should be considered the second-line therapy for ED after failure of PDE5i and should be considered before the indication of penile prosthesis surgery.

## Data Availability

All data generated or analysed during this study are included in this published article

## ABBREVIATIONS

ED: Erectile dysfunction
ICI: Intracavernosal Injection
PDE5i: Phosphodiesterase type-5 inhibitors
NO–cGMP: Nitric oxide–cyclic guanosine monophosphate
PGE1: Prostaglandin E1
ASA: Acetylsalicylic acid
PDDU: Penile Doppler Duplex Ultrasonography
EHS: Erection Hardness Score
IU: International units
mL: milliliters
CC: Cubic centimeters
